# The Effect of Internet-Based Cognitive Behavioral Therapy (I-CBT) for Severe Fatigue in Adolescents with Immune Dysregulation Disorders: Preliminary Findings using a Multiple Single-Case Experimental Design

**DOI:** 10.1007/s10880-025-10096-y

**Published:** 2025-10-22

**Authors:** Linde N. Nijhof, Anouk Vroegindeweij, Jan Houtveen, Hans Knoop, Joris M. van Montfrans, Patrick Onghena, Elise M. van de Putte, Sanne L. Nijhof

**Affiliations:** 1 Department of Pediatrics, Wilhelmina Children’s Hospital, University Medical Centre Utrecht, HP KE.04.133.1, P.O. Box 85090, 3508 AB Utrecht, The Netherlands; 2 Department of Pediatric Immunology and Rheumatology and Infectious Diseases, Wilhelmina Children’s Hospital, University Medical Centre Utrecht, Utrecht, The Netherlands; 3 Department of Medical Psychology, Amsterdam University Medical Centres, University of Amsterdam, Amsterdam Public Health Research Institute, Amsterdam, the Netherlands; 4 Faculty of Psychology and Educational Sciences, KU Leuven, Louvain, Belgium

**Keywords:** Fatigue, Immune dysregulation disorder, Cognitive behavioral therapy, Adolescent, Pediatric rheumatic diseases

## Abstract

Severe fatigue in adolescents with immune dysregulation disorders (IDD) is prevalent and affects daily functioning. This study tested Internet-based cognitive behavior therapy (I-CBT) for the treatment of persistent fatigue in adolescents with IDD. This study used a multiple single-case experimental design (SCED) with a randomized waiting list period of 7–26 weeks, followed by 26 weeks of I-CBT and a 16-week follow-up. Nine adolescents participated, focusing on fatigue severity as the primary outcome. Secondary outcomes included physical functioning, school absence, and, if applicable, pain severity. Pre–post-differences were examined across subjects and individually for weekly measures. At the group level, pre–post-differences in primary and secondary outcomes were examined using mixed models. The across-subject analyses demonstrated the expected effect for fatigue severity. Furthermore, there was a significant reduction in fatigue severity and school absence, and improvement in physical functioning. Single-case analyses demonstrated significant reductions in fatigue in 5/9 patients post-I-CBT, with improvements in physical functioning noted in 6/9 patients. Effect sizes ranged from medium to large. Pain severity significantly improved in 1/5 patients with a small effect size. In a sample of nine adolescents, results indicated that I-CBT could be an effective and feasible treatment of persistent severe fatigue in IDD.

**Supplementary Information:**

The online version contains supplementary material available at 10.1007/s10880-025-10096-y.

## Introduction

Severe fatigue is a common and burdensome symptom in adolescents diagnosed with an immune dysregulation disorder (IDD), with prevalence rates ranging from 18.6 % to 25.1% (Nap-van der Vlist et al., 2019; Nijhof et al., 2016, 2021). The patient group IDD includes all rare immunological disorders such as auto-immune diseases, pediatric rheumatic diseases, auto-inflammatory disorders, and primary immunodeficiency disorders. Severe fatigue in adolescents with IDD is associated with lower Health-Related Quality of life (HRQoL) and significant limitations in daily functioning and is therefore a clinically relevant problem (Nijhof et al., 2016, 2021). An association between fatigue in IDD and disease activity or pharmaceutical interventions, such as the use of Methotrexate (MTX), seems plausible. However, it is important to note that fatigue persists even in adolescents whose disease is in remission, suggesting the involvement of other contributing factors (Nijhof et al., 2016, 2021; Ringold et al., 2013). Given these observations, the persistence of fatigue is likely multifactorially determined, warranting consideration of modifiable factors beyond disease-specific ones. In addition, fatigue can be seen in a broader perspective that affects the adolescent's development. For example, Dowsett and Colby (1997) and Rangel et al. (2000) have noted that persistent severe fatigue during adolescence can disrupt key developmental milestones, with potentially long-term consequences. Adolescence is a critical period for physical, cognitive, emotional, and social growth. Fatigue during this time can hinder academic progress, limit social interactions, and interfere with the development of independence and coping skills. If left untreated, these disruptions may lead to cumulative limitations, such as reduced educational attainment, weakened peer relationships, and prolonged functional impairment into adulthood. In light of these potential risks, the timely treatment of persistent severe fatigue among adolescents is paramount and is crucial to prevent long-term adverse consequences for development (Dowsett & Colby, 1997; Rangel et al., 2000).

To our knowledge, there is no evidence-based intervention for persistent severe fatigue in adolescents with IDD. Randomized controlled trials in adults with different chronic medical conditions have shown that face-to-face and Internet-based cognitive behavior therapy (respectively CBT and I-CBT) can significantly reduce fatigue (Goërtz et al., 2021). The cognitive behavioral model of chronic fatigue assumes that fatigue can be triggered by multiple (disease) factors, such as inflammation or infections, whereas chronic fatigue is also perpetuated by cognitive behavioral factors, such as a disturbed biorhythm or a tendency to catastrophize fatigue (Goërtz et al., 2021). Indeed, fatigue has been associated across various pediatric chronic medical conditions with transdiagnostic, generic behavioral and social factors, which are potentially modifiable (Nap-van der Vlist et al., 2021). Moreover, severe fatigue among adolescents with a chronic condition has repeatedly been found to be related to impediments in daily functioning (Nap-van der Vlist et al., 2019, 2021; Nijhof et al., 2011a, 2011b, 2016, 2021) which decreases after effective treatment of fatigue (Albers et al., 2021; Nijhof et al., 2012). For adolescents, these impediments are expressed in increased school absenteeism and reduced physical functioning. In addition, pain is associated with the severity of fatigue in patients with IDD (Nijhof et al., 2016). Previous research has demonstrated a reduction in pain symptoms during the treatment of chronic fatigue in adolescents, even when the focus of the intervention was not specifically on addressing pain (Nijhof et al., 2012). In the current study, we aim to investigate whether the treatment of fatigue similarly leads to a reduction in pain symptoms in adolescents with fatigue and IDD.

We hypothesized that I-CBT targeting fatigue perpetuating factors could benefit severely fatigued adolescents with IDD. The flexibility of I-CBT, which circumvents school hour conflicts and travel needs, adds to its appeal (Menting et al., 2017). We developed an I-CBT for persistent fatigue in adolescents with IDD named FITNET-plus and tested this intervention with a multiple single-case experimental study design (SCED). A detailed description of the FITNET-plus intervention was recently published in a single-case study (Nijhof et al., 2023). SCED is an approach that can be used to examine treatment effects in small patient groups. This approach provides a cost-effective way to explore whether I-CBT is potentially effective in reducing persistent fatigue among adolescents with chronic medical conditions.

We hypothesized that I-CBT would be associated with a reduction in fatigue. Furthermore, its effects on physical functioning, school absence, and pain were explored. We expected enhanced physical functioning, reduced school absenteeism, and reduced pain severity. Finally, the feasibility of the intervention was examined.

## Patients and Methods

### Study Design and Procedure

An AB phase design was applied in up to ten IDD-diagnosed adolescents. By combining results from repeated experiments, we aimed to show intervention effect using a norm of at least three demonstrations of a treatment effect across patients (Kratochwill & Levin, 2010). One of the included patients was described in depth to enable a thorough introduction of the FITNET-plus intervention (Nijhof et al., 2023).

Phase A represents the no-treatment baseline period, followed by a phase B consisting of 26 weeks of intervention and 16 weeks of follow-up. Both phases employed weekly online self-reported measures for fatigue, physical functioning, and pain. A randomized starting point of the intervention is incorporated as it yields statistical control over time-related variables (Michiels & Onghena, 2019). The study design is illustrated as Fig. 1 in the supplementary material.

The duration of the baseline phase ‘A’ varied from 7 to 26 weeks between patients based on a computer-generated random number list. Once a patient consented to participate in the study, a sealed opaque envelope with a randomly generated duration of Phase A was opened by an independent research assistant in the presence of the patient. After Phase A, patients underwent approximately 26 weeks of I-CBT B followed by 16 weekly follow-up assessments in phase B. Recognizing the time needed to alter habitual responses, we assumed that a patient would improve with a delay in the treatment phase (i.e., a lag). We hypothesized a lag of 18 weeks for fatigue severity, pain severity and daily life functioning based on a study of CBT in Myalgic Encephalitis/Chronic Fatigue Syndrome (ME/CFS) revealing that about three-quarters of the patients benefiting from treatment showed a treatment response around this time span (Heins et al., 2013). Pre-treatment assessment (T0) was combined with a face-to-face intake session to identify the individual perpetuating factors of fatigue. The collected information was used to tailor the FITNET-plus intervention to the individual patients. Six months after the start of the intervention, pre-treatment assessments were repeated in a post-treatment assessment (T1) to explore pre- and post-intervention changes on the group level.

### Study Population and Recruitment

Severely fatigued adolescents with IDD were referred by pediatric rheumatologists and immunologists at Wilhelmina Children’s Hospital (WCH) in the Netherlands. Patients were referred to a social pediatrician from WCH, specialized in chronic fatigue, corresponding to standard care in this hospital. After a uniform diagnostic work-up (via history, laboratory examinations and questionnaires), and a final diagnosis of severe disabling fatigue without a treatable cause by this pediatrician, the patients and their caregivers received brief oral and additional detailed written information about the study. Those willing to participate were further informed about the study by a researcher. Patients and their caregivers gave written informed consent before filling out the questionnaires at T0.

Inclusion criteria for this study were as follows: (a) severe fatigue, defined as a score of ≥ 40 on the fatigue severity subscale of the Checklist Individual Strength (CIS-20) (Vercoulen et al., 1994) for ≥ 3 months (self-reported), (b) age 11.5–18 years , (c) stable medication for 3 months pre-study (type and dosage), (d) self-reported substantial impairment of daily functioning defined as a physical functioning subscale (Child Health Questionnaire, CHQ) (Raat et al., 2002) score ≤ 85% (24), or > 10% school absence in 6 months, and (e) ability to speak, read, and write Dutch and having Internet access. After inclusion, patients were asked not to follow another intervention aimed at fatigue. All interventions to treat the primary disease were continued.

Exclusion criteria were patients with cognitive impairment (IQ < 70), psychiatric co-morbidities that could explain the presence of fatigue or ongoing psychiatric treatment, or somatic causes for fatigue other than IDD. Psychiatric comorbidity was evaluated with validated questionnaires: the State-Trait Anxiety Inventory for children (with a cutoff score ≥ 44) and the Dutch version of the Child Depression Inventory (CDI; with a cutoff score ≥ 15) respectively (Kovacs, 1985; Papay & Spielberger, 1986). Patients who met the criteria for a possible anxiety or depressive disorder were interviewed by a psychologist to make an assessment to determine if the patient was eligible or whether a referral to specialized mental health care was indicated.

### Outcome Measurements

The primary outcome, fatigue, was measured using the CIS-20 *fatigue severity* subscale (8 items, 7-point Likert Scale) with a range of 8–56 and a cutoff score for severe fatigue of 40 (Nijhof et al., 2012).

Secondary outcomes were limitations in functioning, school absence, and, if applicable, pain severity. *Physical functionin*g was assessed weekly with the subscale physical functioning of the validated Dutch translation of CHQ-CF87 (9 items, 0–100%) (Raat et al., 2002), and *school absence* (obliged hours minus attended hours/obliged hours*100%) over the previous two weeks and over the previous 6 months, measured at pre- and post-treatment (Nijhof et al., 2012). *Pain sever*ity was assessed using a weekly Numeric Rating Scale (NRS) ranging from 0 to 10 (respectively no pain – worst possible pain) (Birnie et al., 2019), and patients with NRS ≥ 4 at T0 were monitored weekly during treatment and follow-up.

### Disease Specific Variables and Other Study Parameters

Demographic characteristics were assessed at baseline. Disease- and treatment-related variables were collected from the patients’ medical records by the patient’s physician. Patient ethnicity was determined through self-reported open-ended questions.

Feasibility of the intervention was determined by looking at numbers of patients who were eligible of the total group of referred patients, the willingness to participate with the intervention, the compliance rate of the measurements, and the drop-out rate. After the first intake session, patients completed pre-treatment questionnaires (T0, see Supplementary Table 1) to evaluate the symptom severity, daily limitations, and cognitive behavioral fatigue perpetuating factors. Activity levels were determined for consecutive twelve days and nights, using a motion-sensing device on the ankle (van der Werf et al., 2000). The pre-treatment assessment was discussed in the second session with patients and parent(s)/caregiver(s) after two weeks.

### Intervention

The FitNet-plus treatment protocol, derived from FitNet (Nijhof et al., 2011a, 2011b), was enriched with evidence-based CBT protocols for adults with chronic health conditions and chronic fatigue (H. J.G. Abrahams et al., 2015; Menting et al., 2017) and with elements suitable for this group based on six clinical interviews with patients (Nijhof et al., 2023). A pain-coping module was added based on these interviews (Nijhof et al., 2023). Medication for IDD continued during I-CBT. In case of disease flare-ups, the patient contacted their rheumatologist or immunologist for advice and treatment. In accordance with the advice of the Ethics Review Board (ERB), the I-CBT paused until remission of the disease flare-up was achieved.

The online FITNET-plus platform consisted of up to eight treatment modules with a total intervention duration of 26 weeks, addressing various fatigue maintaining factors: (1) introduction of I-CBT and psycho-education, (2) goal setting, (3) regulation of the sleep–wake cycle, (4) formulation of helpful fatigue- and illness-related beliefs, (5) activity regulation and graded activity, (6) coping with pain, (7) step-by-step achievement of treatment goals, and (8) relapse prevention. All patients started with psycho-education and goal setting (module 1 and 2). Then they worked on the fatigue perpetuating factors that were applicable to them. Fatigue-related perpetuating factors were identified for each patient. A treatment module was offered (after the waiting period) to a patient if the score on the accompanying questionnaire measuring perpetuating factors was above or under the cutoff point at T0 (Albers et al., 2021; Goërtz et al., 2021) (see also Table 1 in the supplementary material). Examples of these factors include low activity pattern, or catastrophic thinking about fatigue.

Caregivers have access to a dedicated online portal with general treatment information and guidance on how to support their child during each module, available as the modules open. They can email the therapist with questions. For children ≤ 15 years, caregivers often act as coaches, while for those > 15 years, they are encouraged to step back and promote their child's independence in the treatment.

Three trained therapists conducted sessions through an online portal, involving regular e-consults with patients and caregivers. The portal layout for the program was specifically designed for adolescents. A detailed description of the FITNET-plus intervention was recently published in a single-case study (Nijhof et al., 2023).

### Ethical Approval

The study adhered to the Declaration of Helsinki and was approved by Utrecht University Medical Centre's ERB (registration: 16/237, NL56462.041.16).

### Statistical Analysis

#### Group-Level Analyses

The Single-Case Randomization Test (SCRT) was applied across subjects (Michiels & Onghena, 2019) to test for pre–post-intervention differences on the weekly measurements. AB two-phase comparisons were conducted at lag 18 (phase B starts 18 weeks after start intervention) (Heins et al., 2013).

To determine the average level of change before (T0) and after treatment (T1) in fatigue severity, physical functioning, and pain severity for the group (*N* = 9), mixed-model analyses with a random intercept were performed. Mixed-model analysis was also performed to test school absenteeism changes (T0 to T1) across subjects, since increasing school attendance was part of the treatment protocol and thus could not be measured weekly as an effect measure. In accordance with the treatment protocol, systematic and structural accumulation of school hours took place after the physical activity level was sufficiently increased. In the meantime, school hours were deliberately kept to a limited number of hours. This means that weekly measurements did not linearly represent school absenteeism because of fatigue severity. Pairwise comparisons were used to summarize the average level of change in school absenteeism for the group (*N* = 9). The alpha level was corrected for multiple testing using Bonferroni correction.

#### Single-Case Analyses

The permutation distancing test (PDT) was used in testing individual pre–post-differences (Vroegindeweij et al., 2023a, 2023b). This test has more statistical power as compared to single-case SCRT in case of a relatively small number of observations. With 30 to 60 observations, PDT has ≥ 80% power to detect medium treatment effects up to large levels of autocorrelation (Vroegindeweij et al., 2023a, 2023b). The PDT examines whether the patient has similar distributions of the outcome measurement over phases A and B by randomly placing the observations in new orders while adjusting for dependency by maintaining the distance between observations until all possible arrangements of the observed data were created. For each arrangement, the test statistic (mean difference between phases A and B) was determined, and the likelihood of obtaining the observed test statistic or a more extreme value was computed (Vroegindeweij et al., 2023a, 2023b).

Just as for the SCRT at group level, two-phase comparison was conducted at lag 18. Because not all patients may improve at the same effect lag, we also explored individual effect lags. Three independent therapists were asked to analyze the anonymous treatment texts of each patient and indicate when they expected the treatment to have an effect on fatigue severity. Their suggested effect lag was evaluated using the PDT by adapting the start of phase B. A *P* value of less than .05 was considered statistically significant. Single-case effect sizes were computed similar to group-level Cohen’s d that should be interpreted differently due to autocorrelation (small < 1.00, medium 1.00–2.49, and large ≥ 2.50) (Harrington & Velicer, 2015).

## Results

### Study Population

**Fig. 1:**
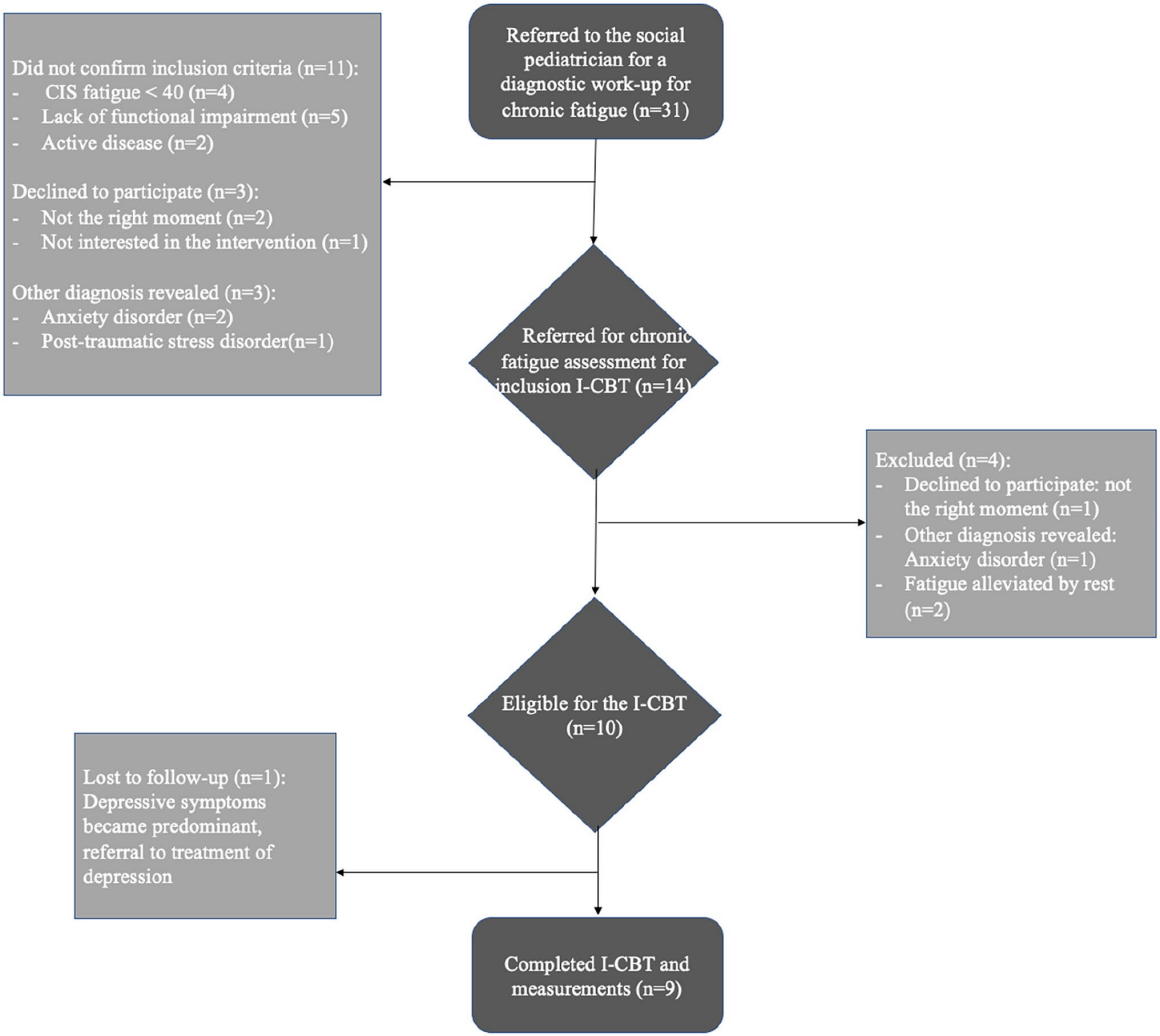
Study flowchart of adolescent patients with IDD and chronic fatigue

Out of 31 referred adolescents between August 2016 and June 2019, 14 (45.2%) were eligible for FITNET-plus pre-treatment assessment. Subsequently, 10 of the 14 patients (71.4%) qualified for treatment, and 9 of them (90%) completed treatment and measurements (see Fig. 1).

### Baseline Characteristics and Compliance

The characteristics of the patients are summarized in Table 1. Baseline characteristics included predominantly female patients (*n* = 7) with European ethnicity (*n* = 7). Two patients were of European/Asian descent. All patients followed designated treatment protocols for their specific auto-immune and immunodeficiency diseases.

**Table 1:** Demographics, number of observations, and compliance per patient

Demographics						Number of observations (weekly measurements)			Compliance of weekly measurements
	Sex	Age (years)	Diagnosis	Disease duration (months)	Fatigue duration (months)	Pre (*n*)	Intervention (*n*)	Follow-up (*n*)	(%)
P1	F	15	JIA (oligoarticular) *Comorbidity: rectal ulcers*	22	22	11/11	22/23	16/16	98.0
P2	M	11	CVID *Comorbidity: none*	58	59	13/13	23/26	4/16	72.7
P3	F	15	JDM *Comorbidity: none*	84	83	13/13	16/26	1/16	54.5
P4	F	18	JIA (polyarticular) *Comorbidity: asthma*	196	15	17/18	24/26	16/16	95.0
P5	M	13	JIA (polyarticular) *Comorbidity:* *microcytic anemia*	63	63	10/13	24/26	13/16	85.5
P6	F	17	Sjogren’s syndrome *Comorbidity:* *celiac disease and IgA deficiency*	95	76	7/7	24/26	16/16	95.9
P7	F	15	JIA (oligoarticular) *Comorbidity: severe constipation*	39	48	8/8	26/26	16/16	100
P8	F	17	JIA (oligoarticular) *Comorbidity: none*	61	29	10/10	28/29	16/16	98.2
P9	F	17	SLE *Comorbidity: none*	24	21	10/10	24/26	15/16	94.2

F = female subjects, M = male subjects; Age = in years, at inclusion, JIA = Juvenile Idiopathic Arthritis, CVID = Common Variable Immune Deficiencies, JDM = Juvenile Dermatomyositis, SLE = Systemic lupus erythematosus (SLE); Disease duration = in months, Pre = weekly assessment period before intervention, Intervention = weekly assessment during intervention phase, Follow-up = weekly assessment during follow-up phase; Compliance is defined as the percentage of delivered notifications that were followed by completing the online weekly questionnaires

### Results for the Weekly Assessments

#### Across Subjects’ Analysis using the SCRT for SCED Data

A significant improvement was found for fatigue severity for effect lag 18 (*p* < .01). Physical functioning and pain severity (outcomes with smaller effects) did not significantly change (data not shown).

#### Single-Case Analysis at 18 Weeks of the Intervention using the PDT for Observational Data

**Fig. 2:**
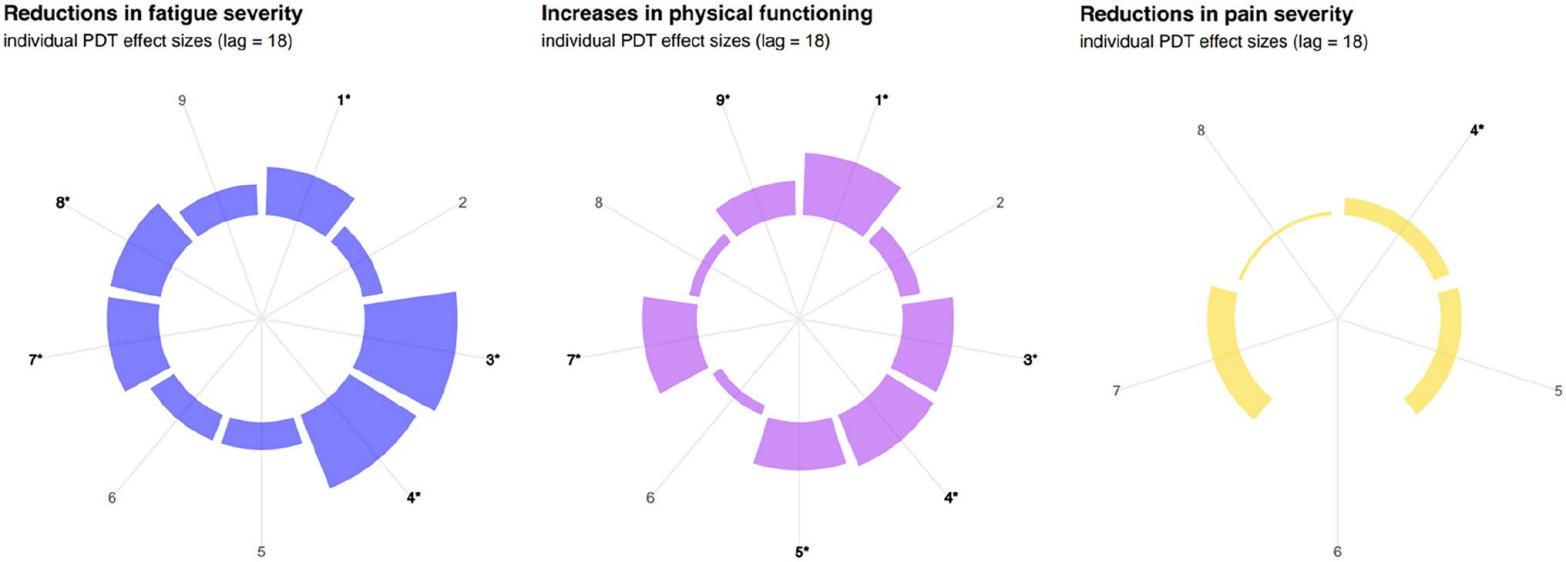
Individual outcomes after FITNET-plus intervention at effect lag 18 expressed in PDT effect sizes

Figure 2 displays the PDT effect sizes and significances for individual patients who followed the FITNET-plus intervention with the assumption of a delayed effect (Effect lag 18). Significant reductions were observed in fatigue severity for five patients. Physical functioning significantly improved in six patients, reaching normal levels for their age and condition. Both fatigue and physical functioning improved with medium to large effect sizes. Pain severity decreased in one patient, with a small effect size. None of the patients deteriorated (significantly) on one of the outcome measures. See supplementary materials for all the single-case graphs with the individual results of all the patients.

#### Exploring Single-Case Treatment Effects at Individual Time Lags

Furthermore, single-case treatment effects at individual time lags were explored, ranging from 5 to 17 weeks as suggested by independent therapists (see Table 2). In contrast to effect lag = 18 weeks, P5 improved significantly in pain severity and P6 improved significantly on pain severity and fatigue severity at effect lag < 18 weeks, which suggests that improvement alternated with relapses at effect lag = 18 weeks. At effect lag < 18 weeks, improvement in physical functioning was not significant in P3, P4, and P7 yet. Their improvement was significant at effect lag = 18, thus suggesting that longer treatment duration was required to establish improvement. In four patients, the amount of improvement at effect lag < 18 was similar to effect lag = 18.

**Table 2:** The *p* values and single-case pre–post-effect size for Lag Time Effects = 18 and explorative analysis for the individual specified Lag Time Effects (1 lag = 1 week) on fatigue severity, physical functioning, and pain severity—for the AB–Phase Design Permutation Distancing Tests on the data of patients 1–9 (P1–P9)

	Lag^a^	Fatigue severity ↓ (CIS)^b^		Physical functioning ↑ (CHQ-87)^c^		Pain severity ↓ (NRS)^e^	
		*p*	Effect size (*d*)^f^	*p*	Effect size (*d*)	*p*	Effect size (*d*)
P1	18	** < .05**	1.4	**.01**	1.8	–	–
	7	** < .05**	1.6	** < .05**	1.7	–	–
P2	18	.06	0.6	.6	-0.1	–	–
	8	.36	0.1	0.668	-0.2	–	–
P3	18	** < .05**	2.7	** < .05**	1.5	–	–
	9	** < .05**	1.1	0.423	0.1	–	–
P4	18	** < .05**	2.3	** < .05**	1.6	** < .05**	0.5
	17	** < .05**	2.1	0.059	1.5	** < .05**	0.6
P5	18	.07	0.8	** < .05**	1.4	0.06	0.6
	9	.12	0.7	** < .05**	1.4	** < .05**	0.8
P6	18	.15	0.8	0.74	−0.3	.47	0
	5	** < .05**	2.2	0.216	0.3	** < .05**	0.9
P7	18	** < .05**	1.5	** < .05**	1.6	.08	0.8
	7	** < .05**	2.4	0.084	1.1	0.149	0.7
P8	18	** < .05**	1.5	0.13	0.3	0.41	0.1
	14	** < .05**	1.9	0.198	0.2	0.536	−0.1
P9	18	0.16	0.9	** < .05**	1.0	–	–
	9	.37	0.4	** < .05**	1.4	–	–

P1–P9 = patients

Note 1. The bold p values represent significance *p* < .05

a: Lag 18 is based on literature about the time of process change of CBT in CFS (Heins et al., 2013)

a: Lag 5–17 is based on the median of the evaluation of three independent therapists (see methods—data analysis for a further description)

b: CIS: Checklist Individual Strength; c: CHQ-87: Child Health Questionnaire; d: school absence per week; e: NRS: Numeric Rating Scale; f: Following the proposed classification for single-case effect sizes similar to Cohen’s d by Harrington and Velicer (2015); effect sizes with a value of 0.00–0.99 are interpreted as small, 1.00–2.49 as medium and ≥ 2.50 as large

### Pre- and Post-Treatment Group-Level Analysis

Table 3 presents the group-level results for fatigue severity, physical functioning, pain severity, and school absence. After following I-CBT, the average improvement in fatigue severity, physical functioning, and school absence was significant, while pain severity did not decrease significantly.

**Table 3:** Group-level analysis pre-treatment (T0) vs. post-treatment (T1)

	Mean pre-treatment	Mean post-treatment	*P*	Mean-difference (95% CI)
Fatigue severity ↓ (scale 8–56)	48.1	29.9	< .001	−18.2 (−26.6 to −9.99)
Physical functioning ↑ (scale 0–100%)	67.1	89.2	.002	22.1 (9.8 to 34.4)
Pain severity ↓ (scale 0–10)	5.0	4.5	.351	−0.5 (−1.6 to 0.6)
School absence, last 6 months ^a^ ↓ (0–100%)	28.3	11.4	.043	−16.9 (−33.1 to −0.7)
School absence, last 2 weeks ↓ (0–100%)	28.9	2.8	.034	−26.1 (−49.9 to −2.4)

CI = confidence interval; M = mean; ↓ = reduced after the intervention, ↑ = increased after the intervention; a = one missing data in post-treatment data. Cutoff score for severe fatigue is 40 or higher (Nijhof et al., 2012), cutoff score for disabilities on physical functioning is 85 or lower (Raat et al., 2002), cutoff score for too much school absence is 10% or higher (Nijhof et al., 2011a, 2011b) and cutoff score for too much pain is 4 or higher (Birnie et al., 2019). Pre–post-analyses were performed with mixed model with a random intercept

### Feasibility of I-CBT for Severe Fatigue in Adolescents with an IDD

The amount of patient eligibility (71.4%), willingness to participate (92.9%), compliance rate of the weekly questionnaires (88.2%), and a low drop-out rate (*n* = 1; due to another predominant diagnosis) indicated that I-CBT was feasible to treat severe fatigue.

## Discussion

This study gives indication for the effectiveness of I-CBT in treating severe fatigue in adolescents with IDD. While group-level analyses, based on the weekly assessments and traditional pre- and post-retrospective questionnaires, showed overall improvement in fatigue severity, physical functioning, and school attendance, individual responses varied. Regarding pain severity, one in five patients reported a reduction, with a small effect size. The intervention was also deemed feasible, and there was no significant worsening of symptoms, supporting its potential use in the treatment of severe fatigue in this population.

These results support I-CBT's utility for adolescents with IDD, aligning with previous research showing its effectiveness in adults with chronic conditions in reducing fatigue severity and improving functioning (Goërtz et al., 2021). There is evidence that similar cognitive behavioral responses to symptoms moderate and mediate treatment outcomes of CBT for fatigue across diverse medical conditions in adults (de Gier et al., 2023). This would imply that patients with different medical conditions may benefit from a transdiagnostic cognitive behavioral approach to fatigue. A recent study in different pediatric populations found that the severity of fatigue symptoms seems to be explained more by transdiagnostic behavioral factors than by disease-specific factors (Nap-van der Vlist et al., 2021). We think it likely on the basis of these findings that severely fatigued adolescents with other chronic conditions could benefit from a transdiagnostic I-CBT aimed at fatigue.

Only a minority of patients in our intervention reported relief from pain symptoms, despite improvements in fatigue. This may be because the treatment focuses specifically on severe fatigue, the primary complaint. Adolescents with severe pain symptoms receive an additional module to prevent pain from hindering fatigue recovery, but FITNET-plus is not designed to directly treat chronic pain. Further research is needed to clarify the relationship between pain and fatigue in patients with IDD and to explore strategies for addressing chronic pain in this population.

The present findings in adolescent patients with IDD and fatigue confirm that I-CBT is a safe treatment, as has been shown previously in other adult populations with a chronic condition (Abrahams et al., 2017; Menting et al., 2017; Nijhof et al., 2012). The adverse events reported in this study were not related to the intervention. One of the inclusion criteria of this study was that patients were in a stable phase of their disease with respect to disease activity and medication use. Two of the nine patients experienced a disease flare-up during treatment, which increased fatigue, pain, and functional impairment. I-CBT was temporarily paused until remission of disease flare was achieved. Both patients were able to resume I-CBT after a short break and consultation with their physician.

Single-case analyses showed that there were differences in patients’ response-time to treatment, the so-called treatment lag. These response times also varied depending on the specific outcome being measured. A possible explanation for these varying response times is that, in our study, patients experienced fluctuating levels of fatigue and daily functioning across weeks. Weekly measurements are more sensitive for individual (disease-related) events, which in turn affected the study outcomes. Pre- and post-treatment measurements showed a clinically relevant improvement for eight of nine patients, and six out of nine patients had a fatigue score below the cutoff level of severe fatigue upon completion of treatment (data not shown). Intervention studies on chronic fatigue typically include a pre-/post-treatment assessment to determine treatment responsiveness and do not provide more detailed assessments of fatigue over time. More research on the variability of fatigue in adolescents would be helpful to further our understanding of this complex and multifactorial symptom.

While I-CBT was effective at the group level, it was not effective for everyone. Some patients might benefit from longer or more intensive therapy or another approach. Despite the FITNET-plus treatment being personalized, with regard to the allocation of the individual treatment modules (Harnas et al., 2021), there is potential for further individualization with regard to individual differences concerning perpetuating factors between patients. A recent study evaluated the data of persistently fatigued adolescents and young adults with various chronic conditions, who completed 28 days of Experience Sampling Methodology (ESM) surveys. The study showed with dynamic single-case networks that there were large inter-individual differences in which biopsychosocial factors were associated with fatigue, in which direction (i.e., positively or negatively associated), and at which timescale (Vroegindeweij et al., 2023a, 2023b). These findings imply that fatigue should be studied at the individual level while taking its dynamic nature into account, and that treatment may be more effective when tailored to the individual’s unique interplay of involved biopsychosocial factors. This prompts the question whether combining an individual ESM study with FITNET-plus therapy could improve the understanding of individual differences in persistent fatigue and the required tailoring of the treatment with consequently higher effectiveness.

Our study had several limitations. Firstly, our study was originally designed as a SCED that should be combined with SCRT analysis both across subjects and single case. A recent study, however, demonstrated that our data can better be analyzed single case with a PDT, since this test has more statistical power for the modest number of single-case longitudinal measurements in our study (Vroegindeweij et al., 2023a, 2023b). However, the PDT is designed for a SCOD, and using this test in our SCED hampers internal validity of single-case results. The results of the current study demonstrated significant pre–post-treatment differences for most patients, which is confirmed by significant differences on the group level. The SCRT analyses on fatigue severity also demonstrated a significant treatment effect across patients, thereby lending greater weight to the conclusions regarding causality in fatigue reduction. In future research, our study design could be replicated with an increased number of longitudinal observations to enable the use of SCRTs to evaluate causal relationships between FITNET-plus and other study outcomes like daily functioning as well. A SCED approach is preferred above an RCT when relatively fewer participants were included. A second limitation is that, because of the intervention’s nature, we were unable to mask the patients or researcher to the treatment. Thirdly, an objective measurement of adherence and of the time patients spent on each treatment’s Web-based module would have improved knowledge of the usability. This approach was not possible because of the technical limitations of the Web portal software. Fourth, covariate analyses were not feasible in the current study due to the characteristics of the data and the specific objectives of the research, although the importance of considering covariates is recognized in many research contexts, and alternative statistical methods that incorporate them could be explored in future studies where appropriate.

In summary, I-CBT, specifically FITNET-plus, shows promise in alleviating severe fatigue and improving functioning in adolescents with IDD. Individualized approaches considering variations in response times and outcomes are crucial for optimizing treatment effectiveness.

## Supplementary Information

Below is the link to the electronic supplementary material.

Supplementary file1 (DOCX 834 kb)

## Data Availability

Data are available from the corresponding author on reasonable request.

## Abbreviations

CBT: Cognitive Behavior Therapy
CHQ: Child Health Questionnaire
CIS: Checklist Individual Strength
I-CBT: Internet-delivered Cognitive Behavioral Therapy
CVID: Common Variable Immune Deficiency
ERB: Ethics Review Board
FITNET: Fatigue In Teenagers on the Internet
HRQoL: Health-Related Quality of Life
IDD: Immune Dysregulation Disorder
JDM: Juvenile Dermatomyositis
JIA: Juvenile Idiopathic Arthritis
ME/CFS: Myalgic Encephalitis/Chronic Fatigue Syndrome
MTX: Methotrexate
NRS: Numeric Rating Scale
PDT: Permutation Distancing Test
PID: Primary Immunodeficiency disorder
PRD: Pediatric Rheumatic Diseases
SCE: Single-Case Experiment
SCED: Single-Case Experimental Design
SLE: Systemic Lupus Erythematosus
SCOD: Single-Case Observational Design
SCRT: Single-Case Randomization Test
