# Supplementary material for: The Effect of Internet-Based Cognitive Behavioral Therapy (I-CBT) for Severe Fatigue in Adolescents with Immune Dysregulation Disorders: Preliminary Findings using a Multiple Single-Case Experimental Design
Source: J Clin Psychol Med Settings. 2025 Oct 22;33(1):119–29. doi: 10.1007/s10880-025-10096-y (PMC13035665; doi:10.1007/s10880-025-10096-y)
Supplement: Supplementary file 1 — Supplementary file1 (DOCX 834 kb) [file 10880_2025_10096_MOESM1_ESM.docx]

| **Supplementary table 1.** Overview of the perpetuating factors targeted for the configuration of the treatment modules in personalized internet-delivered cognitive behavioral therapy (I-CBT) at T0. | | | | |
| --- | --- | --- | --- | --- |
| **Treatment module** | **Targeted perpetuating factor** | **Measured by the following questionnaire(s)** | **Cutoff scores** | **Patients**  **(n)** |
| Helpful thinking regarding fatigue | Dysfunctional cognitions with respect to fatigue | Jacobson Fatigue Catastrophizing Scale (J-FCS, 10-50) (Jacobsen et al., 2004) | ≥ 16 | 9/9 |
|  |  | Self-Efficacy Scale (SES, range 7-28) (JH et al., 1998) | ≤ 19 | 6/9 |
| Helpful thinking regarding pain | Dysfunctional cognitions with respect to pain | Pain Catastrophizing Scale (PCS, range 0-52) (Sullivan et al., 1995) | ≥30 | 1/9 |
| Helpful thinking regarding medical condition | Dysfunctional cognitions with respect to symptoms of the chronic medical condition | Illness Cognition Questionnaire (ICQ) (Kraaimaat et al., 1998) | - |  |
|  |  | Helplessness (range 6-24) |  | 3/9 |
|  |  | Disease benefits (range 6-24) |  |  |
|  |  | Disease acceptance (range 6-24) |  | 3/9 |
| Sleep pattern | Optimizing sleep routine | Diary of bedtimes for 14 days, visual analyses (Nijhof et al., 2011) | - | 9/9 |
| Activity regulation |  | The average physical activity level (number of accelerations per five minute period) measured through actigraphy (van der Werf et al., 2000) |  |  |
|  | Gradually increase physical activities | Low active | ≤1days (activity score ≥66)/12 days (van der Werf et al., 2000) | 3/9 |
|  | First balance between activity as a condition for to build up physical activities and then gradually increase physical activities. | Relatively active | ≥2 days  (activity score ≥66)/12 days (van der Werf et al., 2000) | 6/9 |


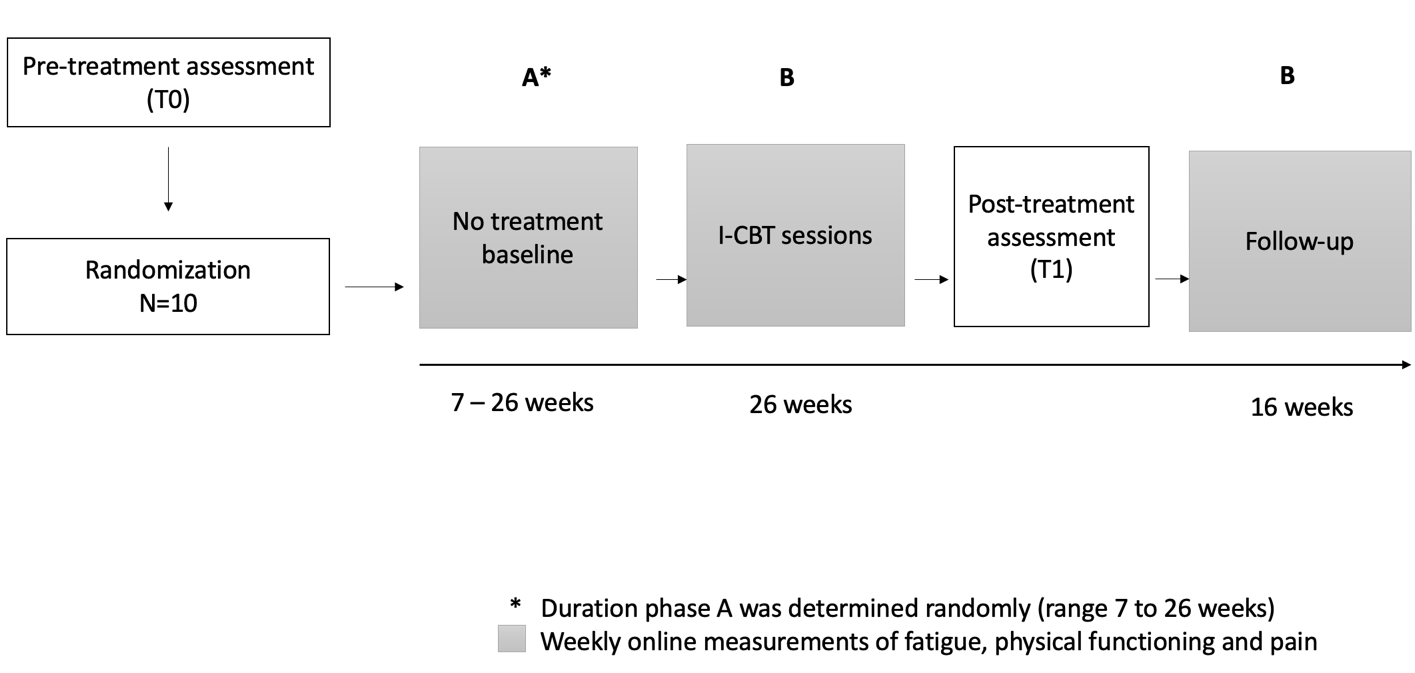


**Supplementary figure 1.** Study design

**The individual results for weekly measurements of fatigue severity (CIS8), physical functioning (CHQ) and pain severity (NRS) – if pain was a treatable factor- across baseline, treatment, and follow-up period of all patients.**

The figures below show the individual results for outcome variables for all patients. All patients received I-CBT, named FITNET-plus. The pre (left side, black) and post (right side, dark gray) treatments values were used in the statistical permutation distancing tests (AB pd-test). The p-value and the pre-post intervention single-case effect size (=d) with an Effect Lag of 18 weeks (phase B starts at the hypothesized moment of improvement: 18 weeks) for the A-B phase design on the raw data are presented.


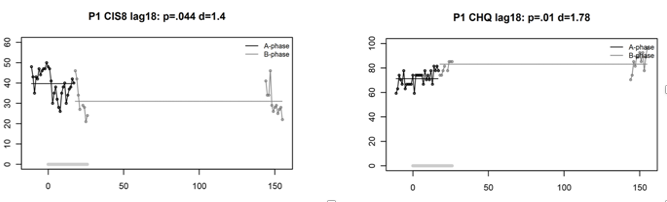


**Supplementary figure 2.** Weekly measurements of fatigue severity (CIS8) and physical functioning (CHQ) across baseline, treatment and follow-up period and the p value and the single-case effect size with an Effect Lag 18 for the A-B phase design permutation distancing tests on the raw data of patient 1 (P1).


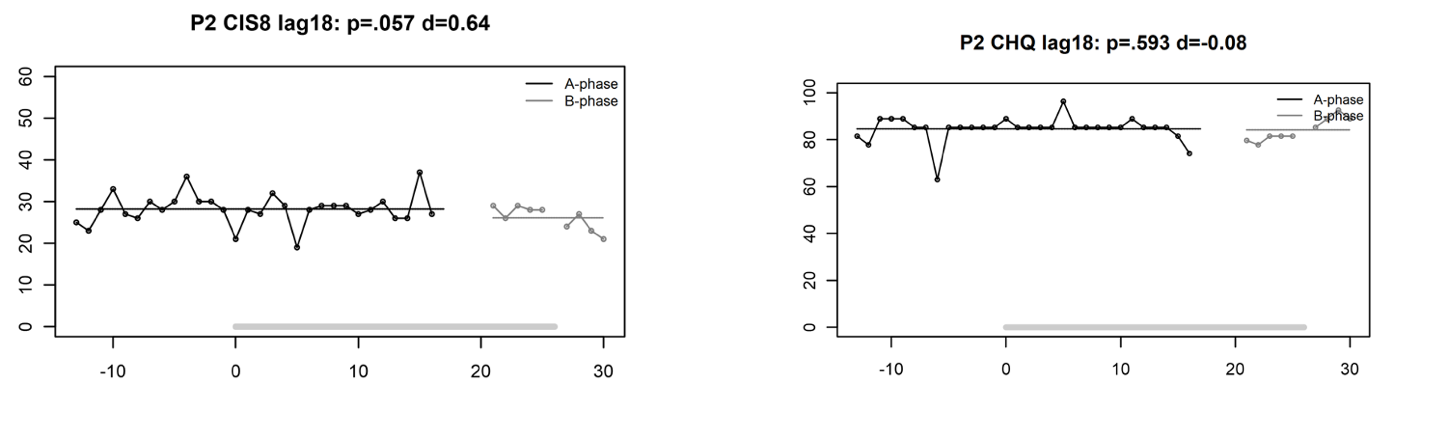


**Supplementary figure 3.** Weekly measurements of fatigue severity (CIS8) and physical functioning (CHQ) across baseline, treatment and follow-up period and the p value and the single-case effect size with an Effect Lag 18 for the A-B phase design permutation distancing tests on the raw data of patient 2 (P2).


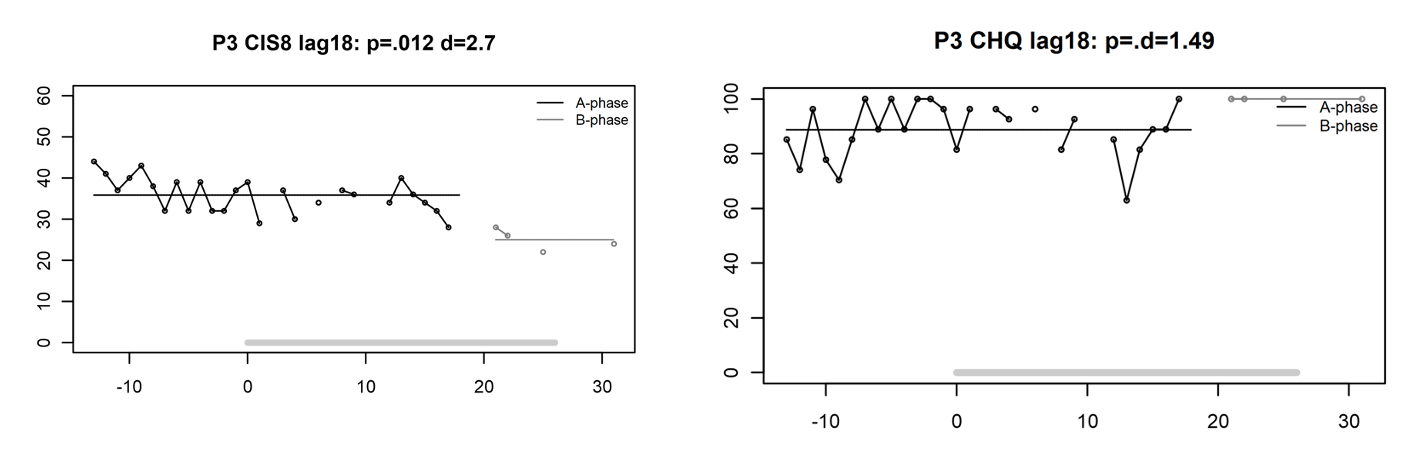


**Supplementary figure 4.** Weekly measurements of fatigue severity (CIS8) and physical functioning (CHQ) across baseline, treatment and follow-up period and the p value and the single-case effect size with an Effect Lag 18 for the A-B phase design permutation distancing tests on the raw data of patient 3 (P3).


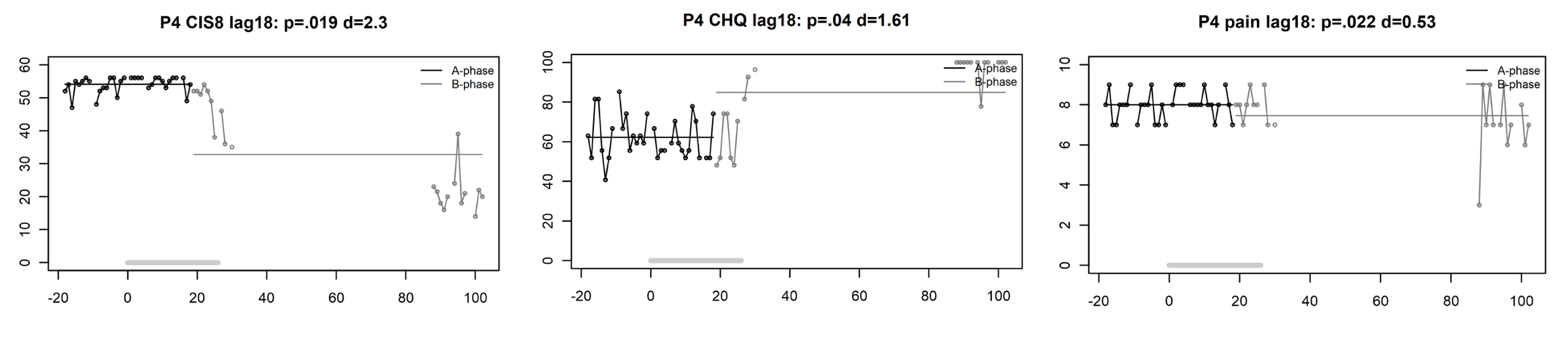


**Supplementary figure 5.** Weekly measurements of fatigue severity (CIS8), physical functioning (CHQ) and pain severity (NRS) across baseline, treatment and follow-up period and the p value and the single-case effect size with an Effect Lag 18 for the A-B phase design permutation distancing tests on the raw data of patient 4 (P4).


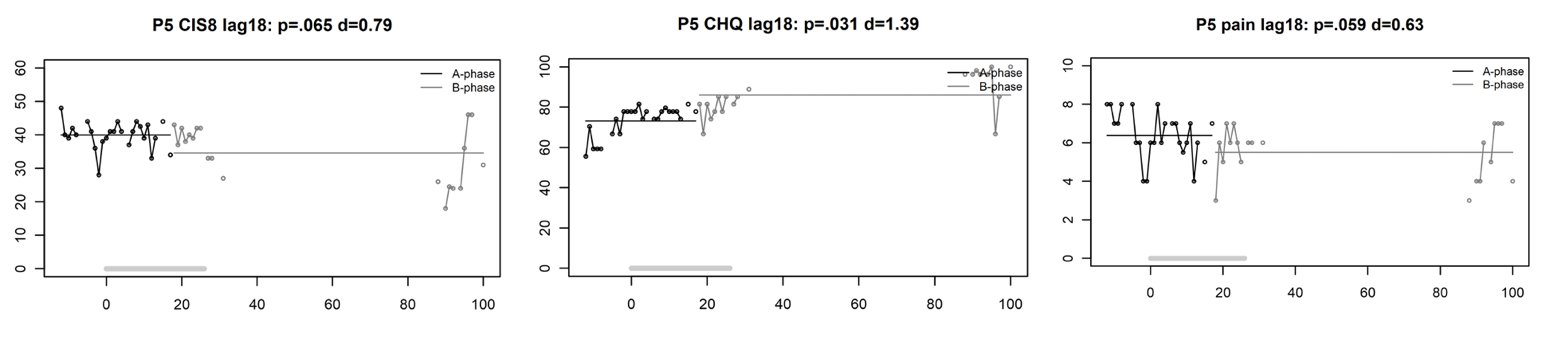


**Supplementary figure 6.** Weekly measurements of fatigue severity (CIS8), physical functioning (CHQ) and pain severity (NRS) across baseline, treatment and follow-up period and the p value and the single-case effect size with an Effect Lag 18 for the A-B phase design permutation distancing tests on the raw data of patient 5 (P5).


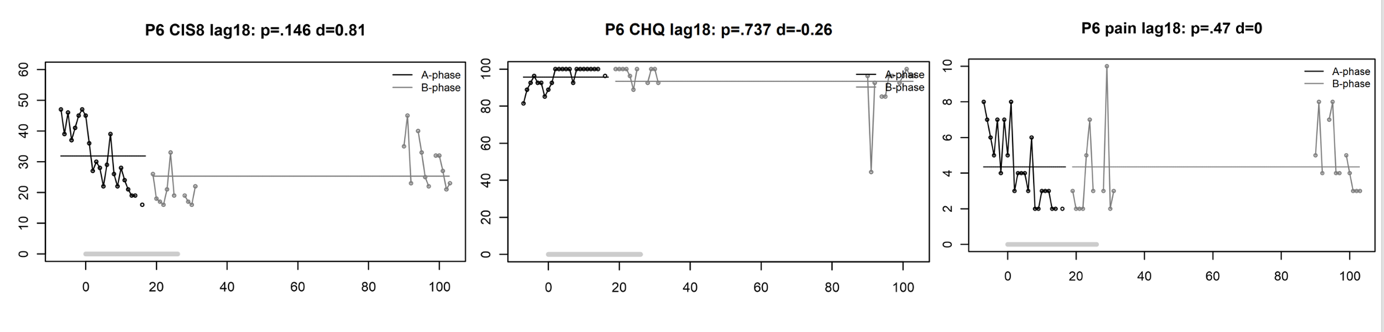


**Supplementary figure 7.** Weekly measurements of fatigue severity (CIS8), physical functioning (CHQ) and pain severity (NRS) across baseline, treatment and follow-up period and the p value and the single-case effect size with an Effect Lag 18 for the A-B phase design permutation distancing tests on the raw data of patient 6 (P6).


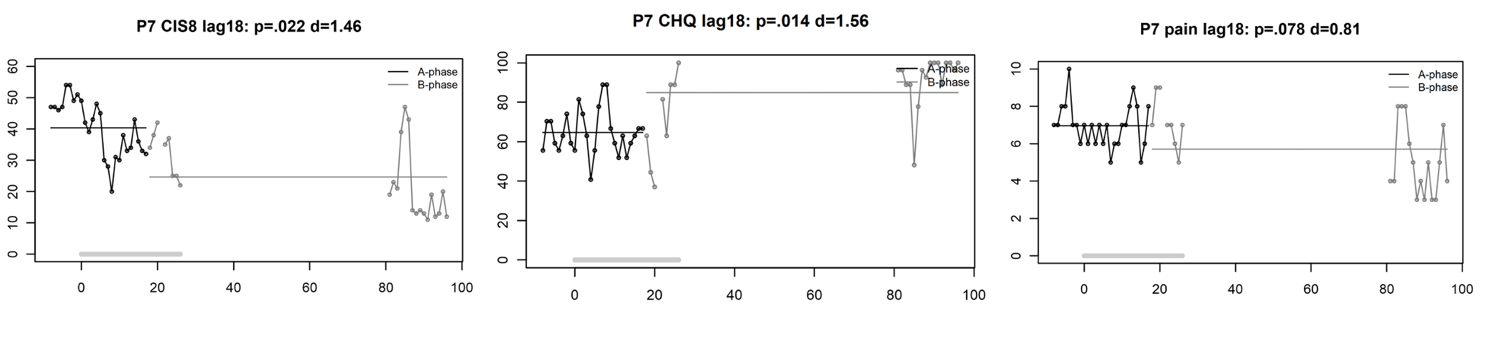


**Supplementary figure 8.** Weekly measurements of fatigue severity (CIS8), physical functioning (CHQ) and pain severity (NRS) across baseline, treatment and follow-up period and the p value and the single-case effect size with an Effect Lag 18 for the A-B phase design permutation distancing tests on the raw data of patient 7 (P7).


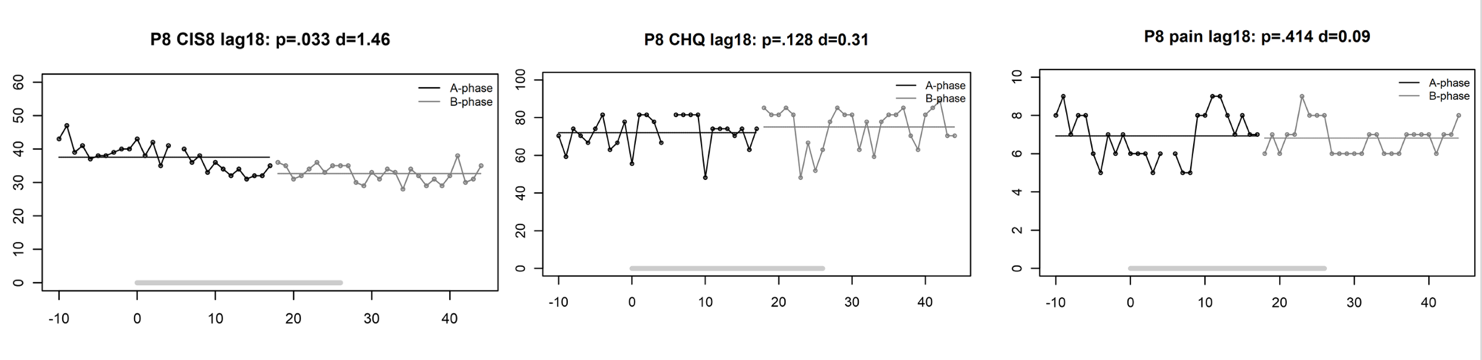


**Supplementary figure 9.** Weekly measurements of fatigue severity (CIS8), physical functioning (CHQ) and pain severity (NRS) across baseline, treatment and follow-up period and the p value and the single-case effect size with an Effect Lag 18 for the A-B phase design permutation distancing tests on the raw data of patient 8 (P8).


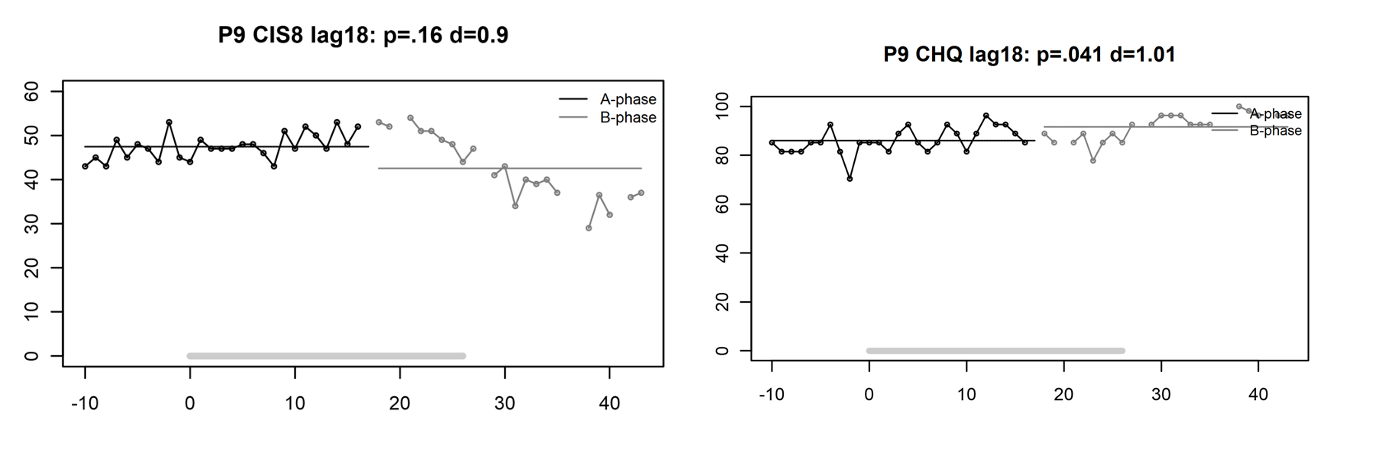


**Supplementary figure 10.** Weekly measurements of fatigue severity (CIS8), physical functioning (CHQ) and pain severity (NRS) across baseline, treatment and follow-up period and the p value and the single-case effect size with an Effect Lag 18 for the A-B phase design permutation distancing tests on the raw data of patient 9 (P9).

**REFERENCES**

Jacobsen, P. B., Andrykowski, M. A., & Thors, C. L. (2004). Relationship of Catastrophizing to Fatigue Among Women Receiving Treatment for Breast Cancer. *Journal of Consulting and Clinical Psychology*, *72*(2), 355. https://doi.org/10.1037/0022-006X.72.2.355

Kraaimaat, Van Lankveld, F. W., Jacobs, W., & Bijlsma, J. W. G. (1998). *Ziekte-Cognitie-Lijst (ZCL) **.

Nijhof, S. L., Bleijenberg, G., Uiterwaal, C. S., Kimpen, J. L., & van de Putte, E. M. (2011). Fatigue In Teenagers on the interNET--the FITNET Trial. A randomized clinical trial of web-based cognitive behavioural therapy for adolescents with chronic fatigue syndrome: study protocol. [ISRCTN59878666. *BMC Neurology*, *11*(1), 23. https://doi.org/10.1186/1471-2377-11-23 [doi]

Sullivan, M. J. L., Bishop, S. R., & Pivik, J. (1995). The Pain Catastrophizing Scale: Development and Validation. *Psychological Assessment*, *7*(4), 524–532. https://doi.org/10.1037/1040-3590.7.4.524

van der Werf, S. P., Prins, J. B., Vercoulen, J. H., van der Meer, J. W., & Bleijenberg, G. (2000). Identifying physical activity patterns in chronic fatigue syndrome using actigraphic assessment. *Journal of Psychosomatic Research*, *49*(5), 373–379. https://doi.org/S0022-3999(00)00197-5 [pii]

Vercoulen, J.H., Swanink, C.M., Galama, J.M., Fennis, J.F., Jongen, P.J. , Hommes, O.R., van der Meer, J.W., Blijenberg, G. (1998). The persistence of fatigue in chronic fatigue syndrome and multiple sclerosis: development of a model. *Journal of Psychosomatic Research*, *45*(6), 507–517. https://doi.org/10.1016/S0022-3999(98)00023-3
